# Polymorphisms of *ACMSD*-*TMEM163*, *MCCC1*, and *BCKDK*-*STX1B* Are Not Associated with Parkinson's Disease in Taiwan

**DOI:** 10.1155/2019/3489638

**Published:** 2019-01-02

**Authors:** Kuo-Hsuan Chang, Chiung-Mei Chen, Yi-Chun Chen, Hon-Chung Fung, Yih-Ru Wu

**Affiliations:** Department of Neurology, Chang Gung Memorial Hospital, Chang Gung University College of Medicine, Taipei, Taiwan

## Abstract

Previous genome-wide association studies in Caucasian populations suggest that genetic loci in amino acid catabolism may be associated with Parkinson's disease (PD). However, these genetic disease associations were limitedly reported in Asian populations. Herein, we investigated the effect of top three PD-associated genetic variants related to amino acid catabolism in Caucasians listed on the top risk loci identified by meta-analysis of genome-wide association studies in PDGene database, including aminocarboxymuconate-semialdehyde decarboxylase- (*ACMSD-*) transmembrane protein 163 (*TMEM163*) rs6430538, methylcrotonyl-CoA carboxylase 1 (*MCCC1*) rs12637471, and branched-chain ketoacid dehydrogenase kinase- (*BCKDK-*) syntaxin 1B (*STX1B*) rs14235, by genotyping 599 Taiwanese patients with PD and 598 age-matched control subjects. PD patients demonstrate similar allelic and genotypic frequencies in all tested genetic variants. These ethnic discrepancies of genetic variants suggest a distinct genetic background of amino acid catabolism between Taiwanese and Caucasian PD patients.

## 1. Introduction

Parkinson's disease (PD) is an age-related neurodegenerative disease with a high rank in prevalence, accounting for 1% of individuals over the age of 65 [1]. The major symptoms include tremors, rigidity, bradykinesia, and stooped posture, which are due to progressive loss of nigrostriatal dopaminergic neurons with presence of eosinophilic cytoplasmic inclusion bodies (Lewy bodies) enriched with *α*-synuclein [1]. Currently, the details in molecular pathogenesis remain unclear. The discoveries of mutation in alpha-synuclein (*SNCA*), parkin *(PARK2)*, PTEN-induced putative kinase 1 (*PINK1*), *DJ-1*, leucine-rich repeat kinase 2 (*LRRK2*) ATPase type 13A2 (*ATP13A2)*, vacuolar protein sorting-associated protein 35 (*VPS35*), eukaryotic translation initiation factor 4 gamma 1 (*EIF4G1*), synaptojanin-1 (*SYNJ1*), dnaJ (Hsp40) homolog, subfamily C, member 6 (*DNAJC6*), and dnaJ (Hsp40) homolog, subfamily C, member 13 (*DNAJC13*) in early-onset PD improve our understanding of potential pathogenesis of PD [2], as well as proposed pathogenic mechanisms such as accumulation of misfolded proteins, mitochondrial dysfunction, oxidative stress, impairment of ubiquitin-proteasome, autophagy-lysosome, and mitophagy [2]. In addition, genome-wide association studies (GWAS) for PD also revealed genetic associations linked to other pathways such as neurotransmission, vascular pathology, transcriptional dysregulation, neuroinflammation, and amino acid metabolism [3, 4].

Pathways in amino acid metabolism, particularly in tryptophan [5, 6] and branched-chain amino acids [7, 8], may contribute to PD pathogenesis. Several genetic variants potentially involved in amino acid metabolic pathways, such as aminocarboxymuconate semialdehyde decarboxylase- (*ACMSD-*) transmembrane protein 163 (*TMEM163*) rs6430538, methylcrotonyl-CoA carboxylase 1 (*MCCC1*) rs12637471, branched-chain ketoacid dehydrogenase kinase- (*BCKDK-*) syntaxin 1B (*STX1B*) rs14235, were listed on the top risk loci by meta-analysis of GWAS in Caucasian [3, 9]. However, these genetic associations in Han Chinese are limitedly revealed. To provide more facts about amino acid metabolism genetic loci contributing to PD across different populations, we conducted a case-control study by examining the genotypic and allelic frequencies of *ACMSD*-*TMEM163* rs6430538, *MCCC1* rs12637471, and *BCKDK*-*STX1B* rs14235 in 599 Taiwanese PD patients and 598 control subjects.

## 2. Subjects and Methods

### 2.1. Ethics Statement

This study was performed under a protocol approved by the institutional review boards of Chang Gung Memorial Hospital (ethical license no: 102-5614A3), and all examinations were performed after obtaining written informed consents.

### 2.2. Patient Population

Patients with PD were enrolled from the neurological clinics of Chang Gung Memorial Hospital, Linkou Medical Center. PD patients were diagnosed according to the UK PD Society Brain Bank clinical diagnostic criteria [10]. We also recruited age and ethnic-matched unrelated healthy individuals as control subjects. PD was categorized into early-onset PD (EOPD) with an age at onset of ≤50 years and late-onset PD (LOPD) with an age at onset of 50 years.

### 2.3. Genetic Analysis

Three genetic loci (*ACMSD*-*TMEM163* rs6430538, *MCCC1* rs12637471, and *BCKDK*-*STX1B* rs14235) involved in amino acid metabolism were selected from the risk loci identified by meta-analysis in PDGene database (http://www.pdgene.org/top_results). The single nucleotide polymorphism (SNP) genotyping was performed by Agena MassARRAY platform with iPLEX gold chemistry (Agena, San Diego, CA). By following the manufacture guide, the specific PCR primer and extension primer sequences (Table 1) were designed with Assay Designer software package (v.4.0). 1 *μ*l of the genomic DNA sample (10 ng/*μ*l) was applied to multiplex PCR reaction in 5 *μ*l containing 1 unit of Taq polymerase, 500 nmol of each PCR primer mix, and 2.5 mM of each dNTP (Agena, PCR accessory, and Enzyme kit). Thermocycling was at 94°C for 4 min followed by 45 cycles of 94°C for 20 s, 56°C for 30 s, and 72°C for 1 min than 72°C for 3 min. Unincorporated dNTPs were deactivated using 0.3 U of shrimp alkaline phosphatase. The single base extension reaction was done using iPLEX enzyme, terminator mix, and extension primer mix followed by 94°C for 30 sec followed by 40 cycles of 94°C for 5 sec and 5 inner cycle of 56°C for 5 s and 80°C for 5 sec than 72°C for 3 min (Agena, iPLEX gold kit). After the addition of a cation exchange resin to remove residual salt from the reactions, 7 nl of the purified primer extension reaction was loaded onto a matrix pad of a SpectroCHIP (Agena). SpectroCHIPs were analysed using a MassARRAY Analyzer 4 and the calling by clustering analysis with TYPER 4.0 software.

### 2.4. Statistics

The genotypes of all variants in the PD patients and the controls did not deviate from the Hardy–Weinberg equilibrium. The Pearson's *χ*^2^ test was used to compare allelic and genotypic frequencies between the PD patients and the controls. As this study involved 3 independent genetic loci, we made a modest correction using the Bonferroni method for multiple comparisons with a statistical significance defined at *p* < 0.017.

## 3. Results

A total of 1197 subjects, including 599 PD patients (female/male: 278/321) and 598 control subjects (female/male: 318/280), were recruited. To minimize the effect by the same SNP within the same family, only one proband with familial PD was recruited. The mean age at onset of PD symptoms was 62.53 ± 11.11 years (range 19∼93) and that of control subjects upon recruitment was 59.62 ± 12.66 years (range 17∼89). The allelic (Table 2) and genotypic (Table 3) frequencies of *ACMSD*-*TMEM163* rs6430538, *MCCC1* rs12637471, and *BCKDK*-*STX1B* rs14235 were similar in both PD patients and controls. No statistically significant differences in allelic and genotypic frequencies of all three SNPs between male or female PD (Table 4), EOPD, or LOPD (Table 5) with controls were observed.

## 4. Discussion

The present study shows that *ACMSD*-*TMEM163* rs6430538, *MCCC1* rs12637471, and *BCKDK*-*STX1B* rs14235 are absent of association with PD. It is important to notice that the minor allelic frequency of *ACMSD-TMEM163* rs6430538 in control subjects of our cohort (1.7%) is similar to that (2.5%) in Chinese/Japanese dataset from 1000 Genomes but very different from that (59.2%) in Caucasians of Utah dataset from 1000 Genome (http://www.1000genomes.org/home). *MCCC1* rs12637471 minor allelic frequency (43.2%) in our control subjects is similar to that (41.7%) in Chinese/Japanese dataset but far different from that (75.8%) of Europeans. Minor allelic frequencies of *BCKDK-STX1B* rs14235 in our control subjects (9.6%) and Chinese/Japanese (9.2%) are also different from those (55%) of Europeans. These distinct genetic backgrounds suggest the differential effects of gene loci related to amino acid metabolism on PD risk between Asian and Caucasian populations.

The *ACMSD* encodes aminocarboxymuconate semialdehyde decarboxylase, which prevents the production of quinolinic acid from aminocarboxymuconate semialdehyde [11]. Patients with PD also demonstrate higher plasma level of quinolinic acid compared with controls [6]. The PD-associated loci within or at proximity of *ACMSD*, such as rs640538, rs6710832, and rs6753334, have been reported in the studies from Caucasian populations [3, 4, 9, 12, 13]. Amongst them, rs640538, repeatedly reported by three large studies, could be the most promising PD-associated loci [3, 4, 12]. By contrast, our study found no significant difference in allelic and genotypic frequencies of rs640538 between PD patients and control subjects in Taiwanese population. More association studies will be warranted to clarify the association between rs640538 or other single nucleotide polymorphism and PD in Asian populations.

By catalyzing the carboxylation of 3-methylcrotonyl-CoA to form 3-methylglutaconyl-CoA, MCCC1 is a critical enzyme in leucine catabolism [14]. Consistent with the results in Caucasian, a study in a Chinese cohort showed association between rs12637471 and PD [15]. However, our study failed to replicate the association of rs12637471 with PD risk. The associations between neighboring genetic variants rs1171441/rs1244493050 and PD are also absent in the other Chinese cohort [16]. Branched-chain alpha-ketoacid dehydrogenase (BCKD) complex is an important regulator of valine, leucine, and isoleucine catabolism [17]. *STX1B* encodes a synaptic receptor for vesicle transport [18]. *STX1B* rs8060857 demonstrates potential association with PD risk in Caucasian populations [19], whereas rs4889603 shows a conflict result in Chinese populations [20, 21]. In Caucasian, rs14235 is not only associated with PD risk but also tends to correlate with severity of Lewy body pathology in patient's brains. However, our results did not recapitulate the genetic features in Taiwanese PD patients. Hence more studies are needed to validate the roles of genetic loci of amino acid metabolism in Asian PD populations.

Our study result of genetic disease association in Taiwanese PD is inconsistent with those from other populations, especially from Caucasian. Different sample sizes and genetic heterogeneity among populations may be attributed to the varied findings across different studies. The roles of epigenetic factors and gene-gene interactions have not been evaluated. The potential interactions of unknown environmental factors with these genetic variants on the development of PD have not been explored. More large series of case-control studies in different ethnic populations will be necessary to clarify these results.

## Figures and Tables

**Table 1:** Sequences of primers used for genotyping in this study.

SNP	Forward	Reverse	Extended
*ACMSD*-*TMEM163* rs6430538	ACGTTGGATGTGGCCGCTGTAACTAATCAC	ACGTTGGATGTCAGAGTTCCAACCTCTAGC	TCAAACCTCTAGCACTGTAAGATA
*MCCC1* rs12637471	ACGTTGGATGCATCTTGCCTAGTATCTGCC	ACGTTGGATGCAGGTGAGATTGTGTCACAG	CACACAGAATGCTGTGGCCTTA
*BCKDK*-*STX1B* rs14235	ACGTTGGATGTCCGCTACTTCTTGGACAA	ACGTTGGATGCTCCTCTGTCTCACTTAATG	CAGCGCCAGGTGATG

SNP, single nucleotide polymorphism.

**Table 2:** Comparisons of minor allelic frequencies of single nucleotide polymorphisms (SNPs) between Parkinson's disease (PD) patients and the controls.

Minor allele of each SNP	PD allele, *N* = 1198 (*N*, %)	Controls allele, *N* = 1196 (*N*, %)	OR (95% CI)	*P* value
*ACMSD*-*TMEM163* rs6430538, C	16 (1.3)	20 (1.7)	0.80 (0.41∼1.5)	0.51
*MCCC1* rs12637471, G	511 (42.7)	517 (43.2)	0.98 (0.84∼1.16)	0.84
*BCKDK*-*STX1B* rs14235, G	119 (9.9)	115 (9.6)	1.04 (0.80∼1.37)	0.78

SNP: single nucleotide polymorphism; OR: odds ratio; CI: confidence interval.

**Table 3:** Genetic models of single nucleotide polymorphisms (SNPs) in Parkinson's disease (PD) patients and controls.

SNP	Genotype	PD, *N* = 599 (*N*, %)	Controls, *N* = 598 (*N*, %)	OR (95% CI)	*P* value
*ACMSD*-*TMEM163* rs6430538	TT	584 (97.5)	578 (96.7)		
	TC	14 (2.3)	20 (3.3)	0.69 (0.35∼1.38)	0.30
	CC	1 (0.2)	0		
** **Dominant model	CC + TC vs TT	15 (2.5)	20, (3.3)	0.74 (0.38∼1.46)	0.39
** **Recessive model	CC vs TC + TT	1 (0.16)	0	1.02 (1.00∼1.10)	

*MCCC1* rs12637471	AA	197 (32.9)	192 (32.1)		
	GA	293 (48.9)	295 (49.3)	0.97 (0.75∼1.25)	0.80
	GG	109 (18.2)	111 (18.6)	0.96 (0.69∼1.33)	0.79
** **Dominant model	GG + GA vs AA	402 (67.1)	406 (67.9)	0.97 (0.76∼1.23)	0.77
** **Recessive model	GG vs GA + AA	109 (18.2)	111 (18.6)	0.98 (0.73∼1.31)	0.87

*BCKDK*-*STX1B* rs14235	AA	483 (80.6)	488 (81.6)		
	GA	113 (18.9)	105 (17.6)	1.09 (0.81∼1.46)	0.58
	GG	3 (0.5)	5 (0.8)	0.61 (0.14∼2.55)	0.49
** **Dominant model	GG + GA vs AA	116 (19.4)	110 (18.4)	1.07 (0.80∼1.42)	0.67
** **Recessive model	GG vs GA + AA	3 (0.5)	5 (0.8)	0.60 (0.14∼2.51)	0.48

SNP: single nucleotide polymorphism; OR: odds ratio; CI: confidence interval.

**Table 4:** Comparisons of minor allelic and genotypic frequencies of single nucleotide polymorphisms (SNPs) between female and male Parkinson's disease (PD) patients and the controls.

Minor allele of each SNP	PD (*N*, %)	Controls (*N*, %)	OR (95% CI)	*P* value
Female (allele)	556	636		
** ** *ACMSD*-*TMEM163* rs6430538, C	9 (1.6)	8 (1.3)	1.29 (0.49∼3.37)	0.60
** **TT	270 (97.1)	310 (97.5)		
** **TC	7 (2.5)	8 (2.5)	1.01 (0.36∼2.81)	0.99
** **CC	1 (0.4)	0		
** ** *MCCC1* rs12637471, G	251 (45.1)	277 (43.6)	1.07 (0.85∼1.34)	0.58
** **AA	83 (29.9)	99 (31.1)		
** **GA	139 (50.0)	161 (50.6)	1.03 (0.71∼1.49)	0.88
** **GG	56 (20.1)	58 (18.2)	1.15 (0.72∼1.84)	0.56
** ** *BCKDK*-STX1B rs14235, G	60 (10.8)	61 (9.6)	1.14 (0.78∼1.66)	0.49
** **AA	219 (78.8)	260 (81.8)		
** **GA	58 (20.9)	55 (17.3)	1.25 (0.83∼1.89)	0.28
** **GG	1 (0.4)	3 (0.9)	0.40 (0.04∼3.83)	0.41

Male (allele)	642	560		
** ** *ACMSD*-*TMEM163* rs6430538, C	7 (1.1)	12 (2.1)	0.50 (0.20∼1.29)	0.14
** **TT	314 (97.8)	268 (95.7)		
** **TC	7 ((2.2)	12 (4.3)	0.50 (0.19∼1.28)	0.14
** **CC	0	0		
** ** *MCCC1* rs12637471, G	260 (40.5)	240 (42.9)	0.91 (0.72∼1.14)	0.41
** **AA	114 (35.5)	93 (33.2)		
** **GA	154 (48.0)	134 (47.9)	0.94 (0.66∼1.34)	0.72
** **GG	53 (16.5)	53 (18.9)	0.82 (0.51∼1.30)	0.39
** ** *BCKDK*-*STX1B* rs14235, G	59 (9.2)	54 (9.6)	0.95 (0.64∼1.40)	0.79
** **AA	264 (82.2)	228 (81.4)		
** **GA	55 (17.1)	50 (17.9)	0.95 (0.62∼1.45)	0.81
** **GG	2 (0.6)	2 (0.7)	0.86 (0.12∼6.18)	0.88

SNP: single nucleotide polymorphism; OR: odds ratio; CI: confidence interval.

**Table 5:** Comparisons of minor allelic and genotypic frequencies of single nucleotide polymorphisms (SNPs) between early-onset Parkinson's disease (EOPD) and late-onset Parkinson's disease (LOPD) patients and the controls.

Minor allele of each SNP	PD (*N*, %)	Controls (*N*, %)	OR (95% CI)	*P* value
EOPD (allele)	178	250		
** ** *ACMSD*-*TMEM163* rs6430538, C	3 (1.7)	2 (0.8)	2.13 (0.35∼12.85)	0.40
** **TT	86 (96.6)	123 (98.4)		
** **TC	3 (3.3)	2 (1.6)	2.15 (0.35∼13.11)	0.40
** **CC	0	0		
** ** *MCCC1* rs12637471, G	89 (50.0)	115 (46.0)	1.17 (0.80∼1.73)	0.41
** **AA	27 (30.3)	36 (28.8)		
** **GA	35 (39.3)	63 (50.4)	0.74 (0.39∼1.42)	0.36
** **GG	27 (30.3)	26 (20.8)	1.39 (0.66∼2.89)	0.38
** ** *BCKDK*-STX1B rs14235, G	19 (10.7)	24 (9.6)	1.13 (0.60∼2.12)	0.72
** **AA	70 (78.7)	102 (81.6)		
** **GA	19 (21.3)	22 (17.6)	1.26 (0.63∼2.50)	0.51
** **GG	0	1 (0.8)		

LOPD (allele)	1020	946		
** ** *ACMSD*-*TMEM163* rs6430538, C	13 (1.3)	18 (1.9)	0.67 (0.32∼1.37)	0.27
** ** *TT*	498 (97.6)	455 (96.2)		
** ** *TC*	11 (2.2)	18 (3.8)	0.56 (0.26∼1.20)	0.13
** ** *CC*	1 (0.2)	0		
** ** *MCCC1* rs12637471, G	422 (41.4)	402 (42.5)	0.96 (0.80∼1.14)	0.61
** **AA	170 (33.3)	156 (33.0)		
** **GA	258 (50.6)	232 (49.0)	1.02 (0.77∼1.35)	0.89
** **GG	82 (16.1)	85 (18.0)	0.89 (0.61∼1.29)	0.52
** ** *BCKDK*-*STX1B* rs14235, G	100 (9.8)	91 (9.6)	1.02 (0.76∼1.38)	0.89
** **AA	413 (81.0)	386 (81.6)		
** **GA	94 (18.4)	83 (17.5)	1.06 (0.76∼1.47)	0.73
** **GG	3 (0.6)	4 (0.8)	0.70 (0.16∼3.15)	0.64

SNP: single nucleotide polymorphism; OR: odds ratio; CI: confidence interval.

## Data Availability

The datasets used and/or analysed during the current study are available from the corresponding author on reasonable request.

## Abbreviations

*ACMSD*: Aminocarboxymuconate semialdehyde decarboxylase
*ATP13A2*: ATPase type 13A2
BCKD: Branched-chain ketoacid dehydrogenase;
BCKDK: Branched-chain ketoacid dehydrogenase kinase
*DNAJC6*: dnaJ (Hsp40) homolog, subfamily C, member 6
*DNAJC1*3: dnaJ (Hsp40) homolog, subfamily C, member 13
*EIF4G1*: Eukaryotic translation initiation factor 4 gamma 1
GWAS: Genome-wide association studies
*LRRK2*: Leucine-rich repeat kinase 2
*PARK2*: Parkin
*MCCC1*: Methylcrotonyl-CoA carboxylase 1
PD: Parkinson's disease
*PINK1*: PTEN-induced putative kinase 1
*SNCA*: Alpha-synuclein
SNP: Single nucleotide polymorphism
*STX1B*: Syntaxin 1B
*SYNJ1*: Synaptojanin-1
*TMEM163*: Transmembrane protein 163
*VPS35*: Vacuolar protein sorting-associated protein 35.
